# Understanding educators’ perspectives and experiences of COVID-19 in schools serving children with intellectual/developmental disabilities

**DOI:** 10.3389/feduc.2022.949430

**Published:** 2022-10-31

**Authors:** Erin F. Jones, Vini Singh, Calliope Holingue, Cheryl Lyn Errichetti, Linda Myers, Michael R. Sherby, Christina Gurnett, Jason Newland, Luther Kalb

**Affiliations:** 1 Information Systems Department, Kennedy Krieger Institute, Baltimore, MD, United States; 2 Center for Autism and Related Disorders, Kennedy Krieger Institute, Baltimore, MD, United States; 3 Johns Hopkins Bloomberg School of Public Health, Baltimore, MD, United States; 4 Maryland Center for Developmental Disabilities, Kennedy Krieger Institute, Baltimore, MD, United States; 5 Special Education, Kennedy Krieger Institute, Baltimore, MD, United States; 6 Department of Neurology, Washington University in St. Louis, Baltimore, MO, United States

**Keywords:** teachers, educators, experience, pandemic, intellectual disability, developmental disability, COVID-19 testing, wellbeing

## Abstract

The COVID-19 pandemic has significantly impacted educators, both personally and professionally. However, very little is known about the extent of these impacts among educators’ serving children with intellectual and developmental disabilities (IDD). The present study surveyed 230 educators (teachers, staff, and administrators) to assess their wellbeing, concerns during the pandemic, and perceived importance of various COVID-19 school mitigation strategies. Data were gathered May/June of 2021 from two separate school districts, one in the Midwest and the other in the Mid-Atlantic, serving children with IDD. Nearly half of all survey respondents reported poor wellbeing. Almost all educators reported health of themselves, students, and family members was their greatest concern when compared to pandemic-related disruption of their duties or benefits (e.g., not having enough sick time). Most educators felt disinfecting routines, vaccinations, and daily health checks were the most helpful in preventing the spread of COVID-19 in schools, while in-school mask mandates and weekly testing of students and staff were perceived as less helpful. Our findings suggest that efforts are needed to support the wellbeing of educators during these challenging times. When pandemic-related policies and procedures are decided by administrators, our data suggest educators will review decisions within the framework of health and safety of themselves, their students, and families. Understanding this framework may be particularly valuable when considering implementation of COVID-19 policies, like masking and COVID-19 testing, that are less preferred.

## Introduction

The World Health Organization (WHO) declared the novel coronavirus (SARS-CoV-2) a pandemic on March 11, 2020. The world has since witnessed more than 231 million confirmed cases and the deaths of more than 4.5 million people (World Health Organization, 2021). The COVID-19 pandemic has caused disruptions in nearly every aspect of life and has implications for all settings, including schools and education (Santos and Miguel, 2021; Sherby et al., 2021). While COVID-19 morbidity and mortality are strongly associated with older age, children are also at risk for severe and/or long-lasting outcomes (Delahoy, 2021). Even amongst children who experienced mild or asymptomatic infections, some develop long-term physical, cognitive, and emotional effects that can persist four or more weeks after infection (CDC, 2021d; Kennedy Krieger Institute, 2021). In rare cases, children with COVID-19 have developed multisystem inflammatory syndrome (MIS-C), which is characterized by the inflammation of different body parts, such as the heart, lungs, kidney, brain, skin, gastrointestinal or other organs (CDC, 2020; Northwell Health, 2021; Sick-Samuels, 2021).

Children with intellectual/developmental disabilities (IDD) may be particularly impacted by COVID-19 (Constantino et al., 2020; Kalb et al., 2021; Vasa et al., 2021). The term intellectual and developmental disability describes a group of heterogeneous central nervous conditions, the onset of which occurs during childhood (American Psychiatric Association, 2013; National Institute of Child Health and Human Development, 2021). Children with IDD experience impairments in physical, social–emotional, cognitive, adaptive, language, motor, physical, and/or behavioral functioning (CDC, 2021a). The most common diagnoses reflective of IDD include attention-deficit/hyperactivity disorder (ADHD), autism spectrum disorder (ASD), cerebral palsy, general developmental delay, learning disabilities, and visual or hearing impairment/loss (CDC, 2021a).

Children with IDD are at increased risk for COVID-19 infection due to difficulties in understanding and applying preventive measures, such as physical distancing and masking (Doody and Keenan, 2021). Adhering to COVID-19 mitigation strategies in the school setting is particularly important as social density, coupled with a closed, indoor environment, promotes the spread of respiratory viruses, such as COVID-19 (American Academy of Pediatrics, 2021; CDC, 2021b). If infected, children with IDD are also at increased risk for severe COVID-19 health outcomes due to pre-existing medical conditions (Courtenay and Perera, 2020; Gleason et al., 2021; Kuo and Coleman, 2021; Sherby et al., 2021). For the IDD population, pandemic-related disruptions in services and therapies increase the risk of developmental regression or slowed progress.

COVID-19 outbreaks in the school setting are generally correlated with higher community transmission rates (Russell et al., 2020; Goldhaber et al., 2021; Ismail et al., 2021); however, schools that enforce and practice consistent prevention strategies often see lower rates of transmission (Verlenden, 2021). This is exemplified by a school outbreak in Marin County, California (May–June 2021), where an unvaccinated, and occasionally unmasked, teacher infected half of the children in her classroom (Lam-Hine, 2021). This outbreak emphasizes the importance of school masking and vaccination as an added means of protecting unvaccinated students or those who are not eligible due to age or medical complications.

Mitigation strategies, such as universal masking in schools and vaccinations for eligible individuals, are critical to reducing transmission of COVID-19 in schools and minimizing severe outcomes associated with COVID-19 infections for students and teachers (Delahoy, 2021). These mitigation strategies are especially important given that the Kaiser Family Foundation found that nearly a quarter of teachers (about 1.47 million people) are at higher risk of serious illness from COVID-19 infection due to age or an underlying condition (Claxton et al., 2020). With the primary emphasis on a safe return to in-person education for the benefit of students, educators, and their families and communities, the CDC and American Academy of Pediatrics (AAP) offer detailed guidance for COVID-19 mitigation strategies in K-12 schools (American Academy of Pediatrics, 2021; CDC, 2021b). The current guidance puts a strong emphasis on the use of “layered prevention strategies,” including physical distancing, handwashing, ventilation, contact tracing, cleaning and disinfecting, and universal indoor masking of all students, teachers, staff, and visitors, regardless of vaccination status. The CDC also recommends that fully vaccinated people who have experienced known exposure with someone with suspected or confirmed COVID-19 be tested 5–7 days after exposure, regardless of whether they are exhibiting symptoms (CDC, 2021e). This is especially important since, as of January 20, 2022, many age-eligible U.S. school children are still not fully vaccinated 19% of children ages 5–11 and 54.7% of children ages 12–17; (Mayo Clinic, 2022) and may be more vulnerable to infection, severe complications, and/or outcomes (CDC, 2021b).

Educators, especially those who support children with IDD, have been immensely impacted by the COVID-19 pandemic. The field of education has experienced an increase in attrition and subsequent staffing shortages, already exacerbated by those out sick and/or following quarantine protocols. As a result, many educators are feeling additional stress in an already demanding job, especially those who serve students with IDD (Marshall, 2022). In a survey of educators conducted by the Connecticut Board of Education Union Coalition, 55% of paraprofessionals reported they that have not been able to fully implement their students’ individualized education plan (IEP) and 504 plans as a result of staff shortages, and nearly 70% of respondents felt that their district was failing to balance their professional expectations and their social/emotional needs (Connecticut Board of Education Union Coalition, 2022). While there is ample evidence to show that educators are experiencing high levels of burnout (Kush et al., 2021; Ozamiz-Etxebarria et al., 2021; Kotowski et al., 2022), very little research has examined burnout or mental health issues among educators serving children with IDD.

In addition to burnout and attrition, it is important to understand the full impact of the COVID-19 pandemic on educators. While there is no formal government tracking of COVID-19-related deaths among educators and school employees, there are some resources dedicated to researching, vetting, and documenting these deaths (Education Week, 2020; @ LostToCovid on Twitter, 2021). It is important to note that resources such as these only include mortalities with direct connections to COVID-19 infections and do not determine or indicate where these school personnel contracted COVID-19 (i.e., at school versus in the community). The true infection and mortality rate of educators is grossly underreported, and more established, systematic tracking of COVID-19 infection and mortality rates among educators is needed to fully illuminate the impact of the pandemic upon the field of education.

With the large impact of COVID-19 on teachers and students with IDD, understanding educators’ perceptions of COVID-19 mitigation strategies and experiences within the school context is critical. At present, little is known about this topic, in general, with even less information available from the perspective of educators serving children with IDD. Educator-reported data regarding impacts of the COVID-19 pandemic can help identify predictors of burnout and inform efforts to better support educators, and subsequently the students they serve.

The present study was supported by the National Institutes of Health’s Rapid Acceleration of Diagnostics-Underserved Populations (RADx-UP) program. With a specific focus on underserved communities that have been disproportionately impacted by the COVID-19 pandemic, the RADx-UP program aims to ensure that all Americans have access to COVID-19 testing (RADx, 2020). To date, RADx-UP has funded over 70 community-engaged projects, including the one described in this paper. The goal of the present study is to fill these gaps in knowledge by examining: (1) educator-reported impacts of the COVID-19 pandemic on wellbeing and instruction; and (2) educators’ perceptions of COVID-19 mitigation strategies in two different school districts in the United States. Given the increased burden involved in following COVID-19 precautions in schools, quick school closures and shifts to virtual learning, and increased risk of exposure while at work, we expected teachers and educators to report increased levels of stress and burnout during the COVID-19 pandemic. We also expected an overall educator endorsement of key CDC-recommended COVID-19 mitigation strategies, such as universal masking and routine, asymptomatic testing of students and/or school staff.

## Materials and methods

The present study recruited 230 educators (teachers, staff, and administrators) to participate in this cross-sectional survey-based study from two geographical regions: the Mid-Atlantic (64%) and Midwest (36%; see Table 1 for sample details). Both the Mid-Atlantic and Midwest multi-campus school programs serve children, ages 5 to 21, with a spectrum of developmental, cognitive, speech, and behavioral needs. These supported needs, ranging from mild to severe, include intellectual disability, speech/language impairment, emotional disability, traumatic brain injury, autism, developmental delay, multiple disabilities, and other health impairments. These school settings exclusively serve children with IDD when a child’s local public school cannot meet the needs of their Individual Education Plan (IEP) goals, a federal mandate. Additionally, a third of the students at the Mid-Atlantic school programs are on a certificate of completion track, as opposed to traditional diploma track.

Educators received an anonymous link to complete a brief, online survey from a school administrator between May and June 2021. The survey was administered *via* Qualtrics Survey Platform (Qualtrics, 2018), and consisted of an anonymous questionnaire developed using a combination of items from existing COVID-19 impact scales and new, project-specific items. Participants were offered a $10 gift card reimbursement for their participation; 77% of surveyed educators opted to receive the reimbursement. The survey response rate was 29% (144/500) and 28% (86/303) for the Mid-Atlantic and Midwest, respectively. The local Institutional Review Boards (IRBs) approved all procedures and materials for the study.

## Measures

### Electronic questionnaire

The custom electronic questionnaire collected basic demographic information as well as information about educators’ experiences during the COVID-19 pandemic, such as impacts on their wellbeing, and concerns and perceptions of COVID-19 mitigation strategies in schools, including testing.

### Demographics

Survey demographic variables included chronological age (in years), time spent in the educational field (in years), vaccine status (yes/no), race (classified as White, Black, Asian, American Indian/Alaskan Native, two or more races, Other), Ethnicity (Hispanic/Non-Hispanic), and professional role (see Table 1 for a list of positions).

### COVID impact on teacher wellbeing

Three Likert-scale indicators were used to assess teacher wellbeing. The first was a 4-point scale question about stress (“How would you describe your current level of stress?”; None, Mild, Moderate, Severe). The second was a 5-point scale question assessing burnout (“How would you rate your current level of burnout?”; No Symptoms – I enjoy my work, Occasionally – I am under stress but do not feel burnout, Definitely – Burning out and one or more symptoms, A lot – Symptoms of burnout will not go away and Completely – Need change or some help). The third 5-point scale question assessed the impact on educators’ decision to work in the field (“How have your experiences during the COVID-19 pandemic impacted how you feel about your decision to work in the field of education?”; Strongly regret, Regret, Neutral, Happy, Very Happy).

### Concerns and perception of mitigation strategies

A ranked-choice question with 9 options was used to assess teachers’ pandemic-related concerns (“What are you most concerned about in terms of in-person instruction/school during the COVID-19 pandemic?”). Similarly, another ranked-choice question, with 12 options, was used to explore teachers’ perceptions of COVID-19 mitigations strategies in the school setting (“What strategies do you feel are most helpful to reduce the spread of COVID-19 at school?”). For each of the ranking questions, educators were asked to arrange the options in order of preference (see Table 2).

### Data analysis

Data were examined descriptively and graphically using means, frequency, and percentages. The ranked-choice questions were descriptively reported as continuous (mean rank; lower mean indicated higher preference) and categorical (proportion of times ranked in top three and proportion of times ranked in bottom three) variables. All analyses were performed using R statistical software (R Core Team, 2018).

## Results

### Participants

Survey responses were collected from a total of 230 educators in the Mid-Atlantic (*n* = 148) and Midwest (*n* = 82), and further broken down by role (administrative, teaching, student support, and other; Table 1). While there was a higher proportion of non-teaching staff in the Mid-Atlantic region (49% vs. 26%), the number of teaching staff in each group was fairly matched (76% vs. 61%). The overall sample averaged 12 years working in the educational setting, and a majority identified as teaching staff (60%; *SD* = 8.29 years). The majority of respondents were White (82%) and had at least one dose of a COVID-19 vaccine (89%).

### Staff and teacher wellbeing

Close to half (49%) of the respondents indicated that they were under moderate (41%) to severe (8%) stress at the time of the survey (Table 3). Overall, 43% of respondents reported definite (one or more symptoms) to complete (severe; considering seeking help) burnout. Teachers and staff in the Mid-Atlantic reported higher levels of burnout (48% vs. 33%) than those in the Midwest. With regards to career impact, 12% of respondents regretted their decision to work in the field of education, 53% were neutral and 35% happy. Mid-Atlantic respondents expressed more regret than those in the Midwest (16% vs. 6%; Table 3).

### Staff and teacher concerns

**FIGURE 1:**
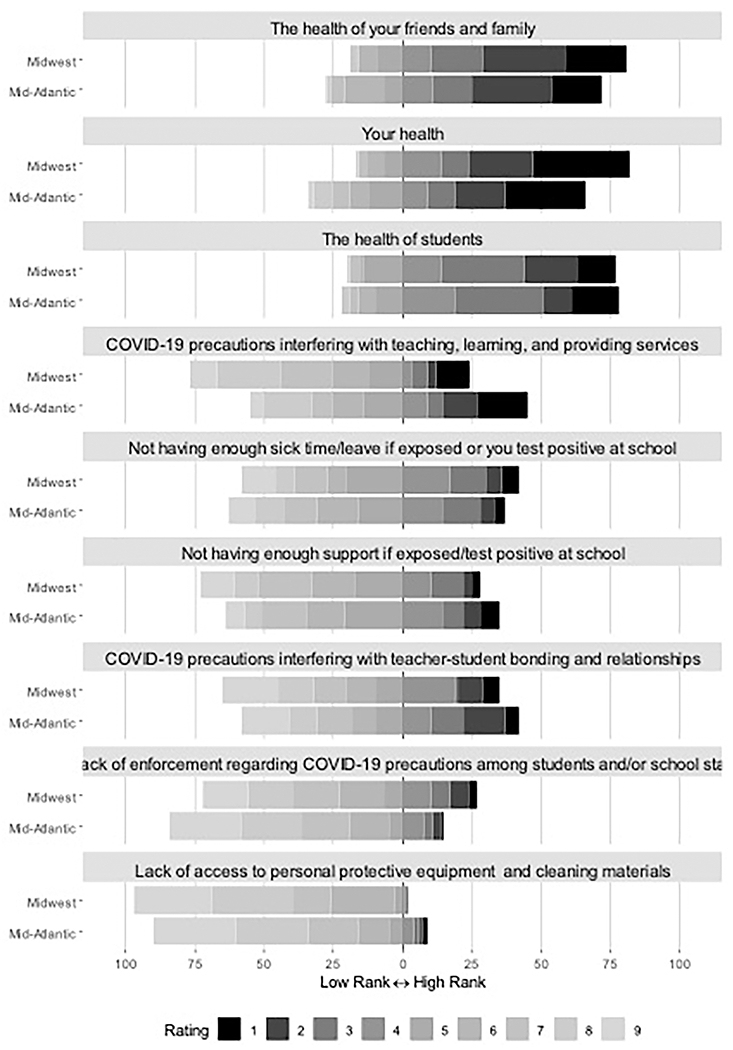
Educator ranking of concerns during the COVID-19 pandemic. Items were ranked in order of which educators were most concerned (1) at the time of survey completion.

As shown in Table 2 and Figure 1, educators indicated that they were most concerned about the health of students, themselves, and others, such as family members and friends. This was followed by COVID-19 precautions interfering with learning and not having enough sick time and support if infected by COVID-19. They were least concerned about the lack of enforcement of COVID-19 protocols followed by lack of access to PPE and cleaning materials.

### Staff and teacher perceptions of mitigation strategies

**FIGURE 2:**
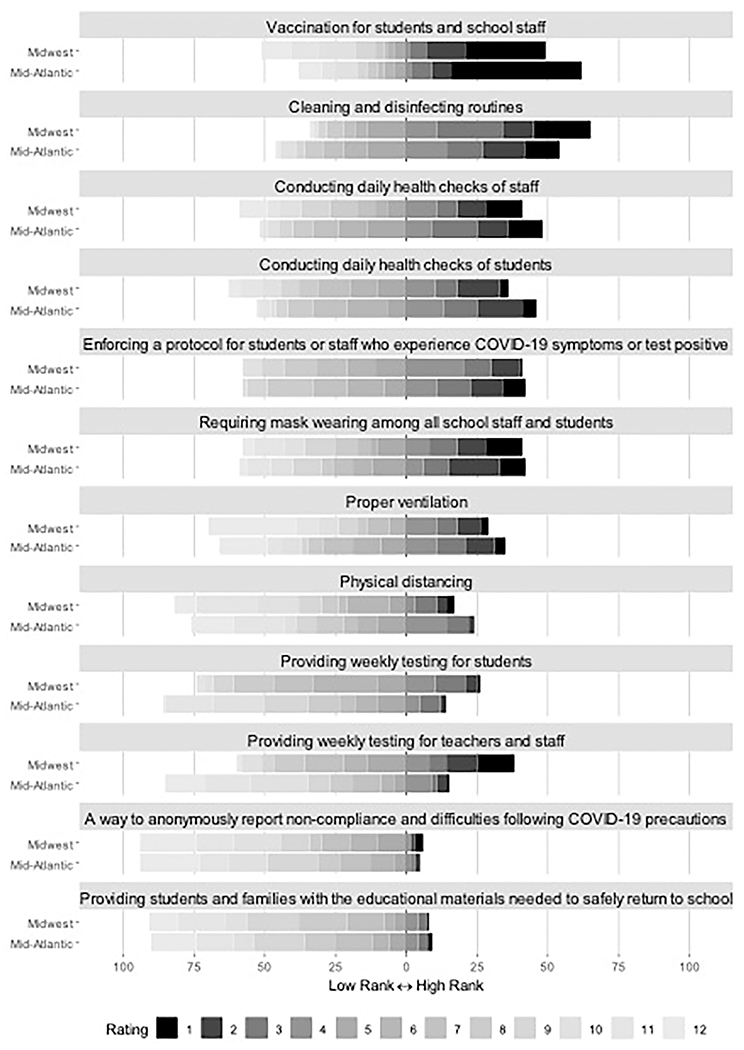
Educators’ perceived helpfulness of COVID-19 mitigation strategies in the school setting. Items were ranked in order of what educators found to be most helpful (1).

As shown in Table 2 and Figure 2, perceived helpfulness of mitigation strategies that prevented the spread of COVID-19 in the school setting were ranked. Most educators ranked cleaning and disinfecting routines, vaccinations, and daily health checks as their top choices. Weekly testing of students and staff was lower on the list. While in-school mask mandates were ranked as more helpful than weekly testing, they too were lower on the list. Weekly testing was more likely to be rated higher in importance in the Midwest sites, and daily health checks were less popular.

**FIGURE 3:**
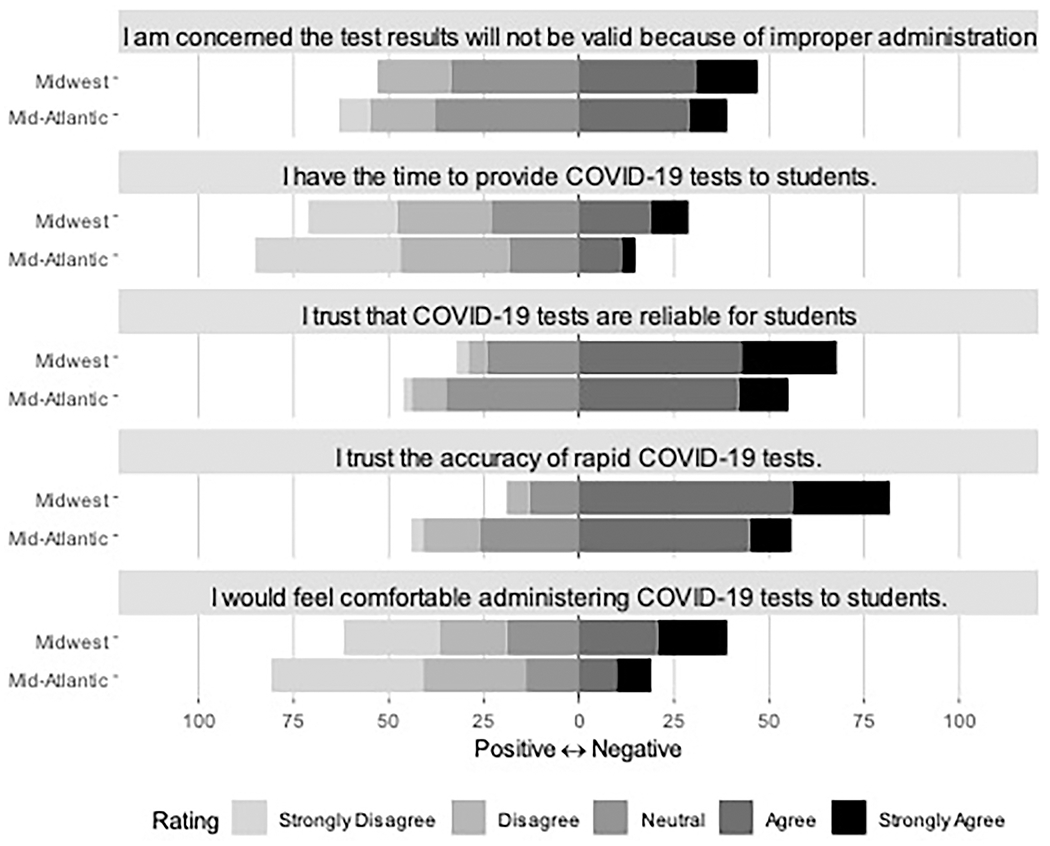
Educator perceptions of COVID-19 testing in the school system. Items rated on a 5-point Likert scale from Strongly Disagree to Strongly Agree.

As seen in Table 2, teachers and staff reported trust in the accuracy of rapid COVID-19 tests overall (65%) and for students specifically (59%). However, the majority of educators also reported that they do not feel comfortable administering COVID-19 tests to students (58%), nor do they feel they have the time to do so (60%). Educators did indicate some concern about the validity of COVID-19 tests administered in schools due to improper administration (42%, Table 2 and Figure 3), and 11% of those in the Mid-Atlantic indicated some distrust regarding COVID-19 test accuracy (Table 2 and Figure 3).

## Discussion

At present, little is known about the impact of COVID-19 on educators serving children with IDD. Across two disparate locations in the US, the present study sought to fill this gap by seeking to understand: (1) the impact of COVID-19 on educators’ wellbeing; and (2) educators’ perceptions of COVID-19 mitigation strategies. Our findings suggest high levels of stress and low levels of provider wellbeing 1 year after the start of the pandemic, although only a few regretted their decision of school employment. Second, we found that educators perceived vaccination, disinfecting routines, and daily health checks of students and staff as being more helpful in preventing the spread of COVID-19 in schools than distancing and weekly testing of students, teachers, and staff. Additionally, while educators generally reported that they trust the reliability and accuracy of COVID-19 tests, they are not comfortable administering the tests themselves, nor do they feel they have the time in their day to do so.

Throughout the COVID-19 pandemic, educators have experienced substantial stress and burnout, which likely explains an increase in rates of teacher attrition (Carver-Thomas et al., 2021; Santos and Miguel, 2021). The few studies that have directly investigated teacher mental health during the COVID-19 pandemic indicate high reports of mental health concerns in this population (Kush et al., 2021). For instance, a study by Ozamiz-Etxebarria et al. found significant percentages of surveyed teachers who reported suffering from stress (50%), anxiety (49%), and depression (32%) as a result of COVID-19 pandemic impacts (Ozamiz-Etxebarria et al., 2021). Consistent with existing reports of educator burnout, we found that many educators reported some level of burnout with their job, and almost all reported feeling stressed. Only 1 in 3 reported that they are happy with their career choice, with most respondents reported feeling neutral about their job choice. Per CDC guidance, schools should consider providing resources for their educators to assist with mental health wellbeing and related coping (CDC, 2021c, “Measures”).

Surprisingly, we found that teachers did not prioritize weekly testing of students and staff in comparison to other COVID-19 prevention methods like cleaning and disinfecting. This may be because a majority of educators did not feel comfortable administering COVID-19 tests to students, nor do they feel they have enough time to administer tests during the school day. This is not wholly unexpected given the many responsibilities educators have on a typical school day.

These findings are critical to consider in light of the National Institutes of Health’s Rapid Acceleration of Diagnostics-Underserved Populations (RADx-UP) program. With a specific focus on underserved communities that have been disproportionately impacted by the COVID-19 pandemic, the RADx-UP program aims to ensure that all Americans have access to COVID-19 testing (RADx, 2020). To date, RADx-UP has funded over 70 community-engaged projects, including the one described in this paper. One of the focuses of the RADx-UP initiative is to increase school-based asymptomatic COVID-19 testing for both students and school staff. However, the present study has experienced challenges with on-site testing enrollment, which is likely a result of educators not having the time or feeling they have the competency to administer COVID-19 tests to students. Our finding that weekly student and staff testing is of less priority for educators is a critical insight for the RADx-UP initiative, which has invested hundreds of millions of federal dollars in this effort. It also has direct implications for future pandemic-related efforts, focused on school-based testing (Haroz et al., 2021; Sherby et al., 2021).

Now that we are aware of educators’ reluctance to administer COVID-19 screening tests to students, future investigations should explore the motivation behind these responses. Aside from not having time, other reasons may include fear of additional exposure, self-confidence in test administration, modality of test administration (e.g., saliva versus nasal), impacts on teacher-student relationships, and/or personal beliefs. Better understanding the motivation behind these responses can shed light on specific areas of direction for future research.

We also found educators’ ranked mask-wearing lower in terms of priority when compared to other prevention strategies. While mask-wearing is part of CDC guidance on the reduction of COVID-19 in general, especially in school settings, it can be a particularly contentious political topic. Educators in this study ranked mask-wearing as less helpful in preventing the spread of COVID-19 compared to other infection control strategies such as vaccination, cleaning and disinfection, and daily health checks. However, the Ohio Schools COVID-19 Evaluation Final Report reported that, when asked if they felt that masks worn consistently and correctly stop the spread of COVID-19, 77.9% of teachers surveyed answered: “a lot” or “completely” (The Ohio Schools COVID-19 Evaluation Research Team, 2021). While many schools have incorporated universal masking as part of their COVID-19 risk mitigation strategy, the perceived effectiveness, and the level to which these strategies are followed is largely unknown.

Despite the importance, to the authors’ knowledge, this is some of the first data regarding teacher-reported perceptions of COVID-19 mitigation strategies in schools. Interestingly, educator feedback in this study does not necessarily align with mitigation strategies recommended for schools. It is possible that clear, consistent communication around mitigation strategies has not occurred for educators, particularly given the contentious political climate surrounding many components of COVID-19 safety measures (e.g., masking, vaccination). Speculation aside, it is important to understand the implications of this misalignment to better support teachers, while ensuring consistent enforcement of data-informed recommendations.

This study had several strengths. Foremost, this study fills an important gap in the scientific literature regarding COVID-19 impacts on educators’ mental health and wellbeing, particularly those serving children with IDD. Additionally, there is scant literature featuring teacher-reported perceptions of COVID-19 mitigation strategies in schools. This study also included a relatively large sample, including two groups in the Mid-Atlantic and Midwest which allowed for greater generalizability.

This study is not without its limitations. There was likely selection bias given the lower response rate, which impacted the ability of this study to confidently assess relationships between stress/burnout and other educator variables (e.g., age). Unfortunately, no data are available on the educators who received the survey invitation but did not participate in completing the survey. It is also difficult to fully generalize the findings in this study, as the sample includes participants from only two school districts. Those sites also had uniquely different experiences prior to the survey (e.g., community infection rates, routine testing had been in place in the Mid-West school district during the prior year). Future studies should include a more diverse sampling from multiple states, and as such, focus on identifying possible geographic-related differences in responses. Additionally, it would be prudent to identify predictors of burnout or stress in educators, especially with regards to variables such as age or years in the field.

In summary, this study found educators across two sites reported low levels of wellbeing during the pandemic. What explains this finding requires further work, with a particular focus on employing qualitative methods. Our findings of educator-reported concerns about their own health and that of their family and students will be an important tool for communicating the importance and continued employment of COVID-19 mitigation strategies in the school setting, especially in light of ongoing revisions of CDC recommendations paired with the common public misperception of the pandemic being an event of the past. Educators are a critical component to child wellbeing, especially those who work with students with IDD. To best support them throughout the ever-changing trajectory of the pandemic, it is essential to thoroughly understand the impact that COVID-19 has had upon teachers and school staff. More established, systematic tracking of COVID-19 infection and mortality rates among educators can help to quantitatively illuminate the impact of the pandemic upon the field of education. Additionally, while schools are provided revised CDC-recommended mitigation strategies, understanding teachers’ experience with these strategies and their perceived effectiveness is important to providing consistent COVID-19 prevention practices in the educational setting. Critical to the intention of collecting teacher-reported perceptions of COVID-19 mitigation practices in schools is the dissemination of such data and findings to further inform policy and practice in the school setting. These measures aimed at improving the support and safety of students, educators, and their families, are essential in the ever-shifting landscape of data-informed pandemic response.

## Figures and Tables

**TABLE 1:** Demographics.

	[ALL]	Mid-Atlantic	Midwest	*N*
		
	*N = 230*	*N = 148*	*N = 82*	
What is your current age? (*M, SD*)	39.4 (11.7)	35.5 (9.82)	46.4 (11.5)	228
How many years have you worked in an educational setting? (*M, SD*)	12.0 (8.29)	9.89 (7.16)	15.9 (8.83)	230
Race: (*N*, %)				226
Black	31 (13.7%)	16 (11.0%)	15 (18.5%)	
White	186 (82.3%)	122 (84.1%)	64 (79.0%)	
Asian	2 (0.9%)	1 (0.7%)	1 (1.2%)	
AIAN	1 (0.4%)	1 (0.7%)	0 (0.0%)	
Two or more races	4 (1.8%)	4 (2.8%)	0 (0.0%)	
Other	2 (0.9%)	1 (0.7%)	1 (1.2%)	
Do you consider yourself Hispanic or Latino? (*N*, %)				228
Yes	7 (3.1%)	6 (4.1%)	1 (1.2%)	
No	221 (96.9%)	141 (95.9%)	80 (98.8%)	
What state do you live in?: (*N*, %)				230
Maryland	143 (62.2%)	143 (96.6%)	0 (0.0%)	
Missouri	75 (32.6%)	0 (0.0%)	75 (91.5%)	
Pennsylvania	3 (1.3%)	3 (2.0%)	0 (0.0%)	
Virginia	1 (0.4%)	1 (0.7%)	0 (0.0%)	
DC	1 (0.4%)	1 (0.7%)	0 (0.0%)	
Illinois	7 (3.0%)	0 (0.0%)	7 (8.5%)	
What is your current job title/role?: (*N*, %)				230
Administrative Staff	27 (11.7%)	21 (14.2%)	6 (7.3%)	
Teaching Staff	137 (59.6%)	76 (51.4%)	61 (74.4%)	
Student Support Staff	58 (25.2%)	44 (29.7%)	14 (17.1%)	
Other Staff	8 (3.5%)	7 (4.7%)	1 (1.2%)	
Have you received the COVID-19 vaccine?: (*N*, %)				228
Yes	203 (89.0%)	133 (89.9%)	70 (87.5%)	
No	25 (11.0%)	15 (10.1%)	10 (12.5%)	

**TABLE 2:** Teacher concerns and attitudes about COVID prevention.

	[ALL]	Mid-Atlantic	Midwest	*N*
		
	*N = 227*	*N = 147*	*N = 80*	

COVID-19 Concerns (*M, SD; lower mean indicates higher priority*)				
Your health	3.24 (2.28)	3.50 (2.42)	2.74 (1.88)	205
The health of your friends and family	3.21 (1.94)	3.37 (2.02)	2.90 (1.74)	205
The health of students	3.34 (1.70)	3.38 (1.72)	3.26 (1.68)	205
COVID-19 precautions interfering with teaching, learning, and providing services	5.11 (2.65)	4.70 (2.65)	5.91 (2.50)	205
Not having enough sick time/leave if exposed or you test positive at school	5.30 (2.21)	5.38 (2.18)	5.14 (2.29)	205
Not having enough support if exposed/test positive at school	5.37 (2.13)	5.19 (2.13)	5.72 (2.09)	205
COVID-19 precautions interfering with teacher-student bonding	5.48 (2.60)	5.30 (2.63)	5.83 (2.52)	205
Lack of enforcement regarding COVID-19 precautions among students and/or school staff	6.61 (2.16)	6.90 (2.02)	6.04 (2.31)	205
Lack of access to personal protective equipment and cleaning materials	7.34 (1.70)	7.29 (1.84)	7.45 (1.39)	205
COVID-19 Mitigation Strategies (M, SD; *lower mean indicates higher priority*)				
Cleaning and disinfecting routines	4.38 (2.72)	4.59 (2.75)	3.97 (2.63)	207
Vaccination for students and school staff	4.81 (4.22)	4.36 (4.10)	5.66 (4.34)	207
Conducting daily health checks of staff	5.30 (3.20)	4.85 (2.84)	6.15 (3.66)	207
Enforcing a protocol for students or staff who experience COVID-19 symptoms or test positive	5.36 (2.54)	5.23 (2.56)	5.62 (2.50)	207
Conducting daily health checks of students	5.48 (2.94)	5.18 (2.76)	6.07 (3.20)	207
Requiring mask wearing among all school staff and students	5.81 (3.49)	5.68 (3.43)	6.06 (3.62)	207
Proper ventilation	7.12 (3.70)	6.65 (3.56)	8.00 (3.82)	207
Providing weekly testing for students	7.26 (2.69)	7.91 (2.68)	6.01 (2.26)	207
Providing weekly testing for teachers and staff	7.41 (3.30)	8.47 (2.96)	5.37 (2.94)	207
Physical distancing	7.80 (3.22)	7.71 (3.24)	7.96 (3.20)	207
Providing students and families with the educational materials needed to safely return to school	8.47 (2.64)	8.45 (2.75)	8.52 (2.45)	207
A way to anonymously report non-compliance and difficulties following	8.81 (2.71)	8.92 (2.58)	8.61 (2.94)	207
COVID-19 Testing Attitudes (n, %)				
I trust the accuracy of rapid COVID-19 tests:				202
Strongly Disagree	4 (2.0%)	4 (3.0%)	0 (0.0%)	
Disagree	24 (11.9%)	20 (15.2%)	4 (5.7%)	
Neutral	43 (21.3%)	34 (25.8%)	9 (12.9%)	
Agree	98 (48.5%)	59 (44.7%)	39 (55.7%)	
Strongly Agree	33 (16.3%)	15 (11.4%)	18 (25.7%)	
I would feel comfortable administering COVID-19 tests to students:				207
Strongly Disagree	71 (34.3%)	53 (39.6%)	18 (24.7%)	
Disagree	49 (23.7%)	36 (26.9%)	13 (17.8%)	
Neutral	33 (15.9%)	19 (14.2%)	14 (19.2%)	
Agree	29 (14.0%)	14 (10.4%)	15 (20.5%)	
Strongly Agree	25 (12.1%)	12 (9.0%)	13 (17.8%)	
I have the time to provide COVID-19 tests to students:				207
Strongly Disagree	68 (32.9%)	51 (38.1%)	17 (23.3%)	
Disagree	57 (27.5%)	39 (29.1%)	18 (24.7%)	
Neutral	41 (19.8%)	24 (17.9%)	17 (23.3%)	
Agree	29 (14.0%)	15 (11.2%)	14 (19.2%)	
Strongly Agree	12 (5.8%)	5 (3.7%)	7 (9.6%)	
I am concerned the test results will not be valid because of improper administration:				207
Strongly Disagree	10 (4.8%)	10 (7.5%)	0 (0.0%)	
Disagree	36 (17.4%)	22 (16.5%)	14 (18.9%)	
Neutral	75 (36.2%)	50 (37.6%)	25 (33.8%)	
Agree	61 (29.5%)	38 (28.6%)	23 (31.1%)	
Strongly Agree	25 (12.1%)	13 (9.8%)	12 (16.2%)	
I trust that COVID-19 tests are reliable for students:				207
Strongly Disagree	4 (1.9%)	2 (1.5%)	2 (2.7%)	
Disagree	16 (7.7%)	12 (9.1%)	4 (5.3%)	
Neutral	64 (30.9%)	46 (34.8%)	18 (24.0%)	
Agree	87 (42.0%)	55 (41.7%)	32 (42.7%)	
Strongly Agree	36 (17.4%)	17 (12.9%)	19 (25.3%)	

**TABLE 3:** Teacher well-being.

	[ALL]	Mid-Atlantic	Midwest	N
		
	*N = 227*	*N = 147*	*N = 80*	
Current level of stress: (*N*, %)				227
None	22 (9.7%)	11 (7.5%)	11 (13.8%)	
Mild	93 (41.0%)	56 (38.1%)	37 (46.2%)	
Moderate	93 (41.0%)	66 (44.9%)	27 (33.8%)	
Severe	19 (8.4%)	14 (9.5%)	5 (6.2%)	
Burnout: (*N*, %)				224
No Symptoms	31 (13.8%)	9 (6.2%)	22 (27.5%)	
Occasionally	98 (43.8%)	66 (45.8%)	32 (40.0%)	
Definitely	63 (28.1%)	44 (30.6%)	19 (23.8%)	
A Lot	21 (9.4%)	18 (12.5%)	3 (3.8%)	
Completely	11 (4.9%)	7 (4.9%)	4 (5.0%)	
Regret about decision to work in education: (*N*, %)				209
Strongly regret	2 (1.0%)	1 (0.8%)	1 (1.3%)	
Regret	22 (10.5%)	18 (13.6%)	4 (5.2%)	
Neutral	112 (53.6%)	76 (57.6%)	36 (46.8%)	
Happy	42 (20.1%)	26 (19.7%)	16 (20.8%)	
Very happy	31 (14.8%)	11 (8.3%)	20 (26.0%)	

## Data Availability

The original contributions presented in the study are included in the article/Supplementary material, further inquiries can be directed to the corresponding author.
